# A sensorimotor paradigm for Bayesian model selection

**DOI:** 10.3389/fnhum.2012.00291

**Published:** 2012-10-19

**Authors:** Tim Genewein, Daniel A. Braun

**Affiliations:** ^1^Max Planck Institute for Biological CyberneticsTübingen, Germany; ^2^Max Planck Institute for Intelligent SystemsTübingen, Germany

**Keywords:** Bayesian model selection, sensorimotor control, structural learning, hierarchical learning, sensorimotor integration

## Abstract

Sensorimotor control is thought to rely on predictive internal models in order to cope efficiently with uncertain environments. Recently, it has been shown that humans not only learn different internal models for different tasks, but that they also extract common structure between tasks. This raises the question of how the motor system selects between different structures or models, when each model can be associated with a range of different task-specific parameters. Here we design a sensorimotor task that requires subjects to compensate visuomotor shifts in a three-dimensional virtual reality setup, where one of the dimensions can be mapped to a model variable and the other dimension to the parameter variable. By introducing probe trials that are neutral in the parameter dimension, we can directly test for model selection. We found that model selection procedures based on Bayesian statistics provided a better explanation for subjects' choice behavior than simple non-probabilistic heuristics. Our experimental design lends itself to the general study of model selection in a sensorimotor context as it allows to separately query model and parameter variables from subjects.

## Introduction

For biological organisms in uncertain environments, at least three important problems arise: one, the estimation of the state from noisy sensory feedback (e.g., the state of body parts). Two, the prediction of sensory consequences of actions and three, the selection of desirable actions, which builds upon the state estimate as well as the capability to predict consequences (Wolpert and Ghahramani, 2000; Todorov, 2004; Shadmehr et al., 2010; Franklin and Wolpert, 2011). Internal models are thought to play a central role in solving these problems (Shadmehr and Mussa-Ivaldi, 1994; Wolpert et al., 1995; Kawato, 1999; Tin and Poon, 2005). For estimation, internal models make use of sensory feedback to update prior beliefs about unobserved variables. Forward models predict sensory consequences of one's own actions, which allows not only to bridge delays in the sensorimotor loop, but also to distinguish between self- and externally generated motion (Poulet and Hedwig, 2006; Imamizu, 2010). To solve the problem of action selection, the theory of optimal feedback control has been used as one of a number of frameworks that study how internal models are harnessed in control (Todorov and Jordan, 2002; Diedrichsen, 2007; Izawa et al., 2008; Braun et al., 2009a; Diedrichsen and Dowling, 2009; Nagengast et al., 2009).

Besides the question of how biological organisms adapt internal models when the environment changes over time, another important question is how they learn new internal models and select between existing models (Shadmehr et al., 2010). There is a large body of evidence that shows that learning of predictive models happens on many different time scales and levels of abstraction (Newell et al., 2001; Smith et al., 2006; Wolpert and Flanagan, 2010). In a number of recent studies (Braun et al., 2009a,b) it was shown, for example, that the motor system can learn structural invariants when faced with randomly changing environments that share a structural similarity. In particular, in these tasks subjects had to both adapt parameters of internal models to environments with known structure and to learn new structures and their parameters from exposure to environments with different variability pattern. Here we are interested in the mechanism by which the motor system selects between different structures, that is the selection between different models that can take on different parameter settings.

In cognitive science, a number of studies has shown that human model selection in categorization or language learning tasks can be well described as Bayesian model selection (Holyoak, 2008; Kemp and Tenenbaum, 2008; Tenenbaum et al., 2011). Bayesian models have also been very successful in explaining human perceptual and sensorimotor learning of parameters in environments with known structure (van Beers et al., 1999; Ernst and Banks, 2002; Knill and Pouget, 2004; Körding and Wolpert, 2004; Körding and Wolpert, 2006; Braun et al., 2009a; Girshick and Banks, 2009). However, if there are several structures, and each structure has a range of parameter values, then the full problem of model and parameter selection arises. For perceptual learning this has been studied, for example, in case of the ventriloquist problem, where subjects have to discriminate whether a visual and an auditory signal stem from one source or from two different sources (Körding et al., 2007; Sato et al., 2007). Here we study Bayesian model selection in the context of a *sensorimotor integration* task that allows for ambiguous stimuli which are compatible with different model classes. The goal of our study is to develop an experimental paradigm for model selection and to test whether human sensorimotor choices in such a setting are quantitatively consistent with Bayesian model selection.

## Results

**Figure 1:**
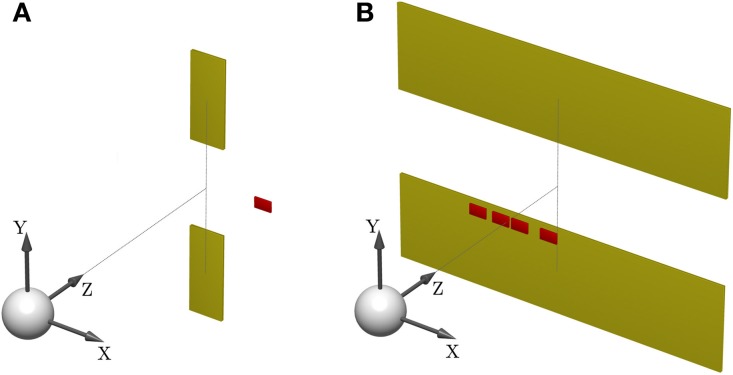
**Sensorimotor task setup. (A)** Shows the schematic of a *standard trial*. One of two targets (yellow) has to be hit after observing a *shifted* cursor (red) for a short feedback duration. The white sphere depicts the start position. **(B)** Illustrates a *probe trial* where the visual feedback is ambiguous and consists of a densely sampled array of cursors, centered horizontally and vertically. The target width covers the whole lateral workspace, in order to make horizontal compensations obsolete. A photograph of the experimental apparatus is provided in Figure 15.

**Figure 2:**
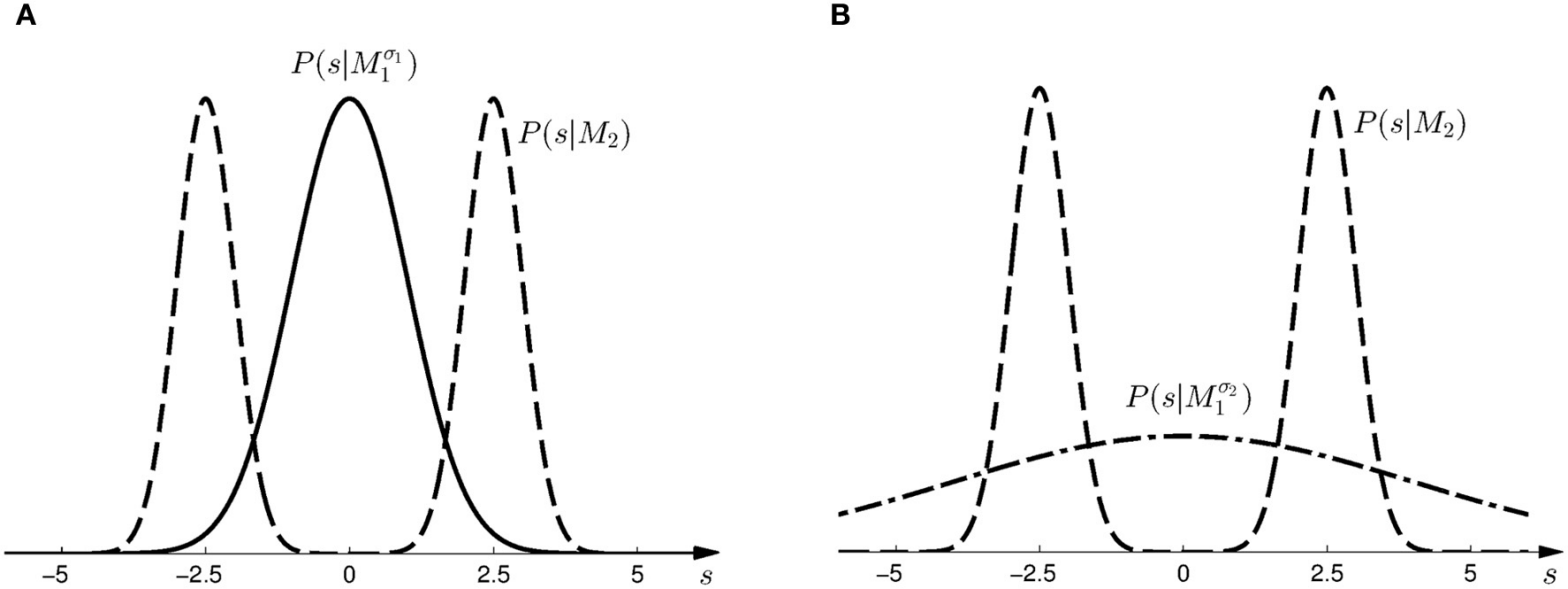
**Prior distributions for the horizontal shift *s*. (A)** Shows the two prior distributions induced by model *M*^σ_1_^_1_ (Gaussian distribution with σ_1_ = 1, solid line) and *M*_2_ (mixture of two Gaussians, dashed line) in the *first part* of the study. **(B)** Shows the two prior distributions induced by model *M*^σ_2_^_1_ (Gaussian distribution with σ_1_ = 4, dash-dot line) and *M*_2_ (same mixture of two Gaussians, dashed line) in the *second part* of the study.

Subjects controlled a cursor from a start position to one of two targets in a 3D virtual reality setup—see Figure 1A. At the start position the cursor was always displayed and represented subjects' veridical hand position. However, during the movement a random lateral shift *s* was applied to the cursor with respect to the hand position. Importantly, throughout most of the movement, the cursor was hidden and there was only a brief time interval of sensory feedback of the shifted cursor position. In each trial, the shift *s* was randomly sampled from one of two possible distributions with 50:50 probability. In the first part of the experiment (first 500 trials), the two distributions were given by a Gaussian *P*(*s*|*M*^σ_1_^_1_) and a mixture of Gaussians *P*(*s*|*M*_2_)—see Figure 2A. In the second part of the experiment (last 500 trials), the standard deviation of the first distribution was increased, so that the two distributions were given by *P*(*s*|*M*^σ_2_^_1_) and *P*(*s*|*M*_2_)—see Figure 2B.

From the point of view of model selection, the two distributions over the shift correspond to two different models *M*_1_ and *M*_2_. Subjects could indicate their choice of model by selecting one of the two targets. Subjects could exploit the observed shift to infer the correct target. Their belief about the shift *s* was reported by a compensatory horizontal movement. In all trials, the upper target represented the selection of model *M*_1_ and the lower target represented the selection of model *M*_2_. After completing a reaching movement, participants were informed about the correctness of their beliefs by showing the shifted cursor and hiding the incorrect target. Subjects were instructed about the relationship between shift and target selection. For example, in the first part of the study they were told that small shifts are mostly associated with the upper target and larger shifts with the lower target—see “Materials and Methods” for details. They could use the first 100 trials of each part of the experiment to acquaint themselves with the decision criterion.

To test for model selection with different degrees of feedback uncertainty, we used *probe trials* where participants were shown ambiguous feedback. The feedback presented in these trials was an array consisting of uniformly and densely sampled rectangles that represented all the possible cursor locations—see Figure 1B. The array width *d* could take on one of three different values with equal probability: small (*d* = 3 cm), medium (*d* = 5 cm), and large (*d* = 8 cm). The larger the array the higher the uncertainty about the cursor position, and therefore the higher the uncertainty about the underlying shift *s*. In these probe trials subjects only reported their belief about the model without indicating the presumed shift. This was achieved by increasing the target width to the full size of the lateral workspace, which made horizontal compensations unnecessary. As in the standard trials, participants reported their belief about the correct model *M* by choosing either the upper or the lower target, however, in probe trials they did not receive any feedback on whether their choice was correct or not. Probe trials occurred intermixed with standard trials after the first 100 trials of each part of the experiment.

**Figure 3:**
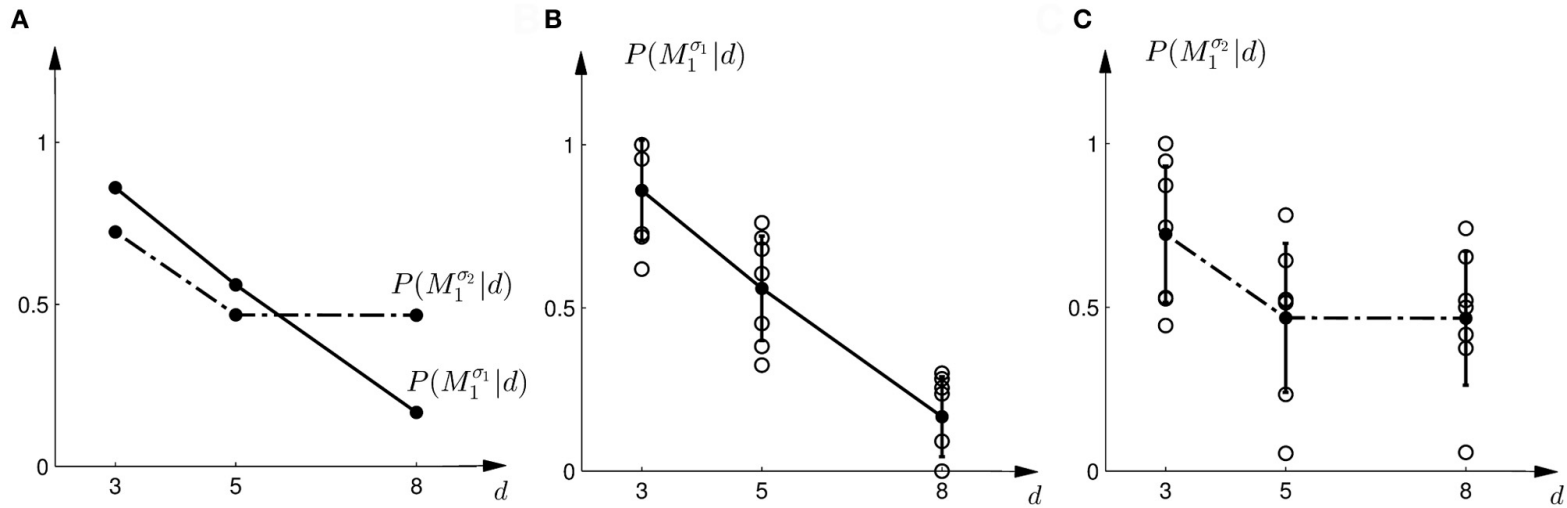
**Subjects' choice behavior in probe trials. (A)** Shows the average of the experimentally observed choice probabilities for choosing *M*_1_ for three different observed array widths *d* of the ambiguous feedback array. The mean is taken over all subjects. The solid line corresponds to the choice probabilities observed in the first part of the study, the dash-dot line shows the choice probabilities observed in the second part of the study, where the variance of *M*_1_ was increased. **(B)** Shows the experimentally observed probability of choosing *M*^σ_1_^_1_. Circles represent individual subjects' choice probabilities, the solid line shows the *average* over participants with standard deviation error bars. With increasing array size, the probability of choosing *M*^σ_1_^_1_ decreases. **(C)** Shows the experimentally observed choice probabilities *P*(*M*^σ_2_^_1_|*d*) for the second part of the experiment, where σ_2_ > σ_1_. Compared to the first part, the probability of choosing *M*^σ_2_^_1_ for the *large* array size *d* = 8 is significantly increased.

In the probe trials of the first part of the experiment we found that the probability of choosing model *M*^σ_1_^_1_ decreases with increasing width of the ambiguous feedback array: for small array widths subjects preferred model *M*_1_, whereas for large ambiguous feedback arrays they preferred model *M*_2_. This can be seen in Figure 3B. The correlation between the array width and the choice probability was statistically significant for all subjects (*p* < 0.01, Fisher's exact test). For the second part of the experiment, subjects' model selection probabilities are depicted in Figure 3C. The correlation between array width and choice probability was no longer significant (*p* > 0.1 for all subjects, Fisher's exact test). This is because, for ambiguous feedback arrays with medium and large uncertainty (*d* = 5, 8 cm) subjects were now indifferent between model *M*_1_ and *M*_2_. Importantly, when comparing the model selection probabilities of the first part of the experiment and the second part of the experiment shown in Figure 3A, the model selection probabilities changed significantly (*p* < 0.05, ranksum test) for ambiguous stimuli with large uncertainty (*d* = 8 cm). For an array width of *d* = 3 or 5 cm there was no significant change in the choice probabilities for the two variance conditions of model *M*_1_ (*p* > 0.05 ranksum test). Importantly, this implies that the choice probabilities of selecting model *M*_2_ are very different for the same stimulus (*d* = 8 cm) depending on the complexity of model *M*_1_.

We tested five different explanatory schemes to describe subjects' choice behaviors: model selection with Bayes factors, model selection with Bayesian policy inference of the discrimination functions learned in the standard trials, and three heuristic explanations that are non-probabilistic.

### Explanation 1: Bayes factors

**Figure 4:**
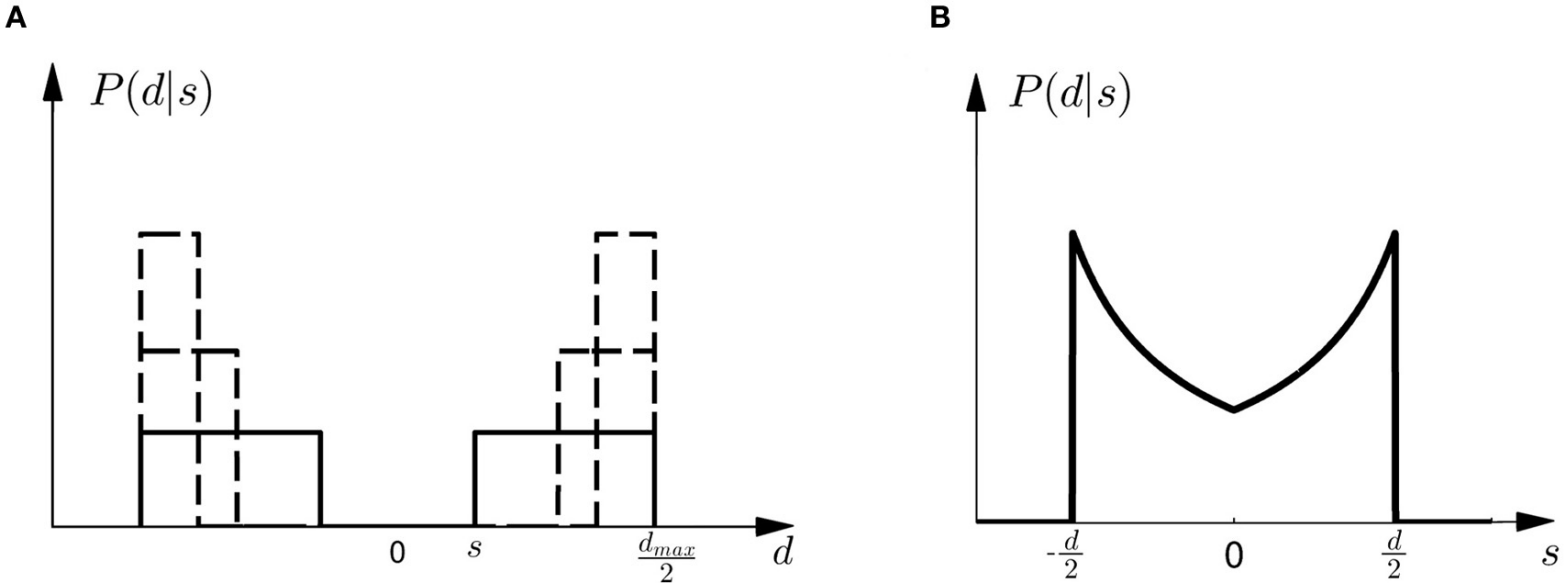
**Observation model. (A)** Shows the probability *P*(*d*|*s*) of observing an array of width *d* given the shift *s*. All array widths up to a maximum *d*_max_ have equal probabilities but arrays that are too small to contain the shift *s* have zero probability. The figure shows three different cases for three different shifts *s* with a solid line and two dashed lines, respectively. It is important to notice that in case of small shifts probability mass is spread quite evenly, whereas in the case of large shifts most of the probability mass is placed close to the maximum array width *d*_max_. **(B)** Shows the likelihood *P*(*d*|*s*) as a function of *s*. For an array width *d* it is more likely that the shift *s* that caused the observation is close to half the array width *d*/2.

Given prior probability *P*(*M*_*i*_) over the two models *M*_1_ and *M*_2_, Bayes' rule describes how to assign posterior probability *P*(*M*_*i*_|*d*) after observing array width *d* in a probe trial, such that *P*(*M*_*i*_|*d*) ∝ *P*(*d*|*M*_*i*_)*P*(*M*_*i*_)—where *P*(*d*|*M*_*i*_) measures how well the array width *d* can be explained on average by the shifts that are compatible with model class *M*_*i*_. This average is also called the *marginal likelihood* or *evidence* and can be computed as *P*(*d*|*M*_*i*_) = ∫ *dsP*(*d*|*s, M*_*i*_)*P*(*s*|*M*_*i*_), where each shift *s* contributes the likelihood *P*(*d*|*s, M*_*i*_) weighted by the prior *P*(*s*|*M*_*i*_) shown in Figure 2. *P*(*d*|*s, M*) is an observation model that explains how likely it is to observe an array of width *d* if the true shift is *s*. In our experiment both models have the same observation model, that is *P*(*d*|*s, M*) = *P*(*d*|*s*), which assigns equal probability to all array widths *d* greater or equal than a given shift *s* up to a maximum width *d*_max_. This uniform distribution over *d* can be seen in Figure 4A for different given shifts *s*. When *P*(*d*|*s*) is used as a likelihood model, however, it is considered as a function of *s* with a particular fixed observation *d*. The likelihood model then indicates how likely all the different shifts *s* would be as an explanation of the observed array width *d*. The likelihood model as a function of *s* can be seen in Figure 4B.

**Figure 5:**
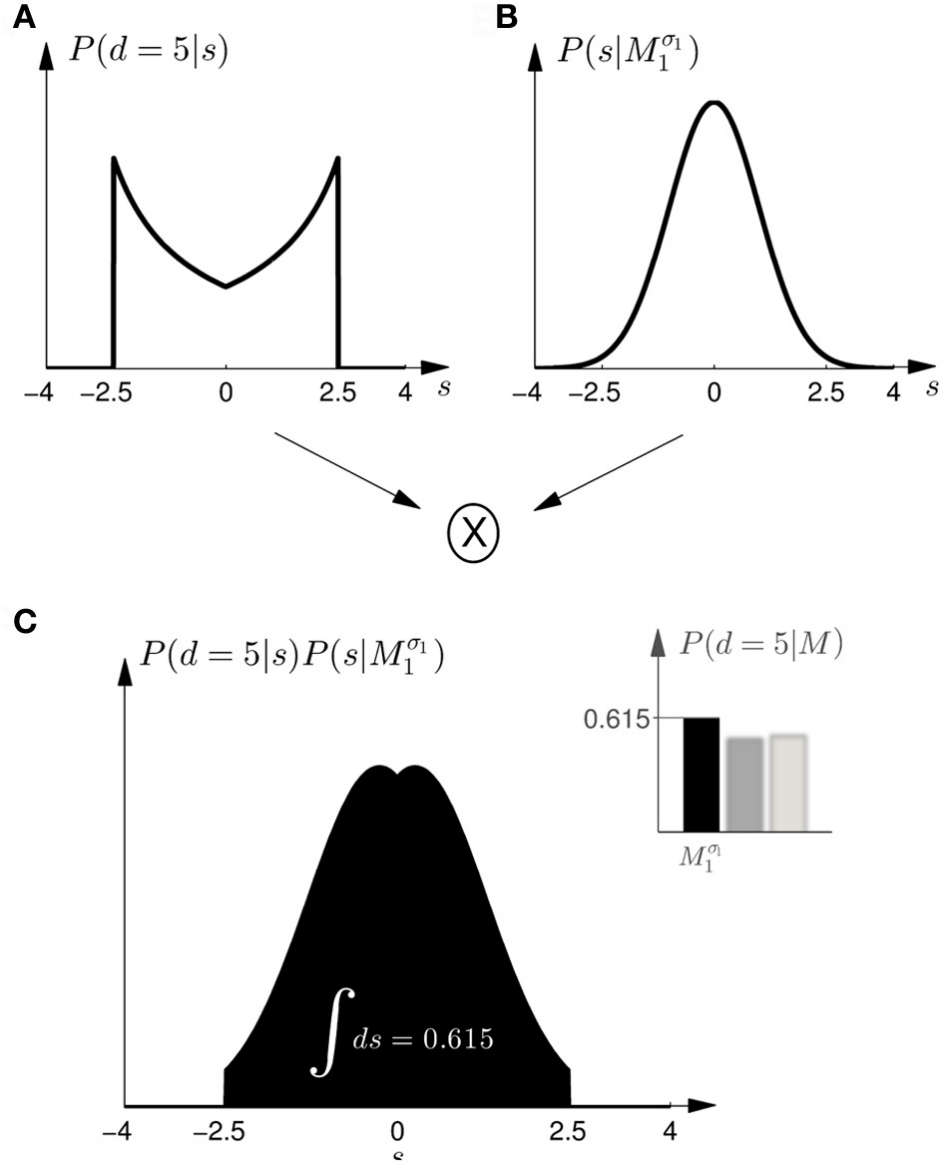
**Computation of the model *evidence P*(*d*|*M*) when observing an ambiguous cursor array of width *d*—shown for model *M*^σ_1_^_1_ and *d* = 5, so the possible shifts of the hand position range from −2.5 to 2.5 cm. (A)** Shows the *likelihood P*(*d* = 5|*s*) of observing the ambiguous cursor array of width *d* = 5 for different shifts *s*. **(B)** Shows the *prior P*(*s*|*M*^σ_1_^_1_) over the shift *s* according to model *M*^σ_1_^_1_. **(C)** Depicts the product of the distributions in panels **(A,B)** and illustrates the integration over the shifts *s*. This computation leads to a single value for the model evidence *P*(*d*|*M*^σ_1_^_1_). The model evidence can be similarly computed for all models as shown in the inlet panel.

**Figure 6:**
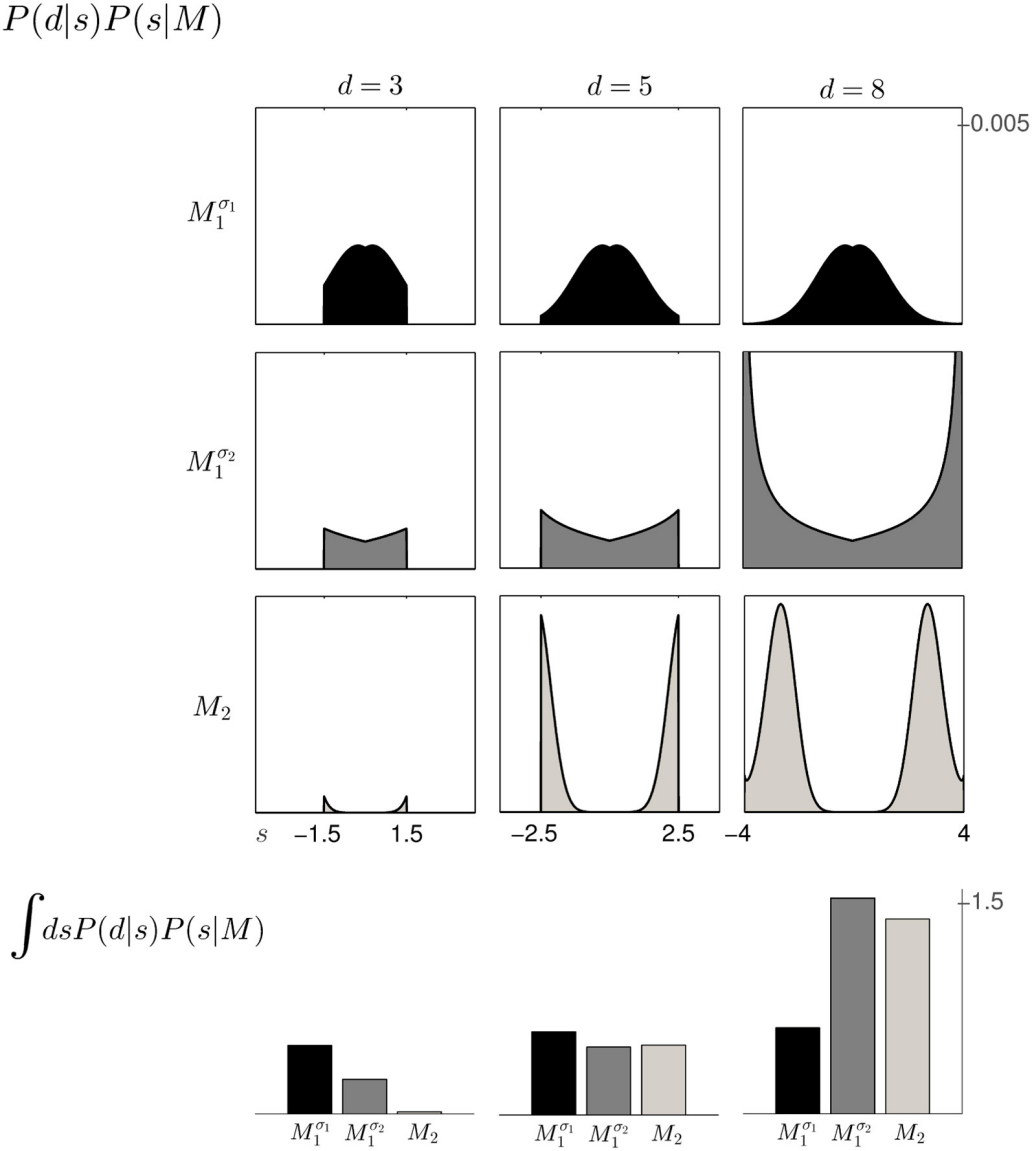
**The 3 × 3 panel shows the product of parameter prior *P*(*s*|*M*) and observation likelihood *P*(*d*|*s*) for the three possible array widths **d**∈ {3, 5, 8} cm and the three different models *M*^σ_1_^_1_ (black), *M*^σ_2_^_1_ (dark gray) and *M*_2_ (light gray).** The **bottom row** shows the *model evidence P*(*d*|*M*) of observing a feedback array of width *d* for the different models, which is obtained by integrating out the shift *s* for each of the three models within the same column. For feedback arrays of large width (*d* = 8 cm), model *M*_2_ is a better explanation than model *M*^σ_1_^_1_. However, when increasing the standard deviation of *M*_1_ to σ_2_ both models have similar marginal likelihood for large width. For a detailed illustration on the computation of the model evidence *P*(*d*|*M*) see Figure 5.

Based on the likelihood model in Figure 4B and the priors shown in Figure 2, Figure 5 explains how the model evidence can be computed for different observations *d*. Figure 6 shows the model evidence for the different widths *d* of the ambiguous feedback array for all three models *M*^σ_1_^_1_, *M*^σ_2_^_1_, and *M*_2_. As can be seen in the bottom row of Figure 6, for small width (*d* = 3 cm) of the ambiguous stimulus array, the evidence for *M*_1_ (for both σ_1_ and σ_2_) is higher than for *M*_2_. This is because model *M*_1_ places a high probability mass on small shifts centered around zero, whereas model *M*_2_ does not—see Figure 2. For ambiguous feedback arrays of medium uncertainty (array width *d* = 5 cm), the evidence of all models is very similar, that means they all can explain a medium-size range of possible shifts equally well. For ambiguous feedback arrays with large uncertainty (array width *d* = 8 cm), the evidence of *M*^σ_1_^_1_ is lower than the evidence for *M*_2_, because model *M*_2_ places more probability mass on larger feedback array widths, which ultimately results from the higher probability placed on large shifts—see Figure 6. However, when the standard deviation of model *M*_1_ is increased to σ_2_ in the second part of the study, both models can explain ambiguous feedback with large uncertainty equally well.

**Figure 7:**
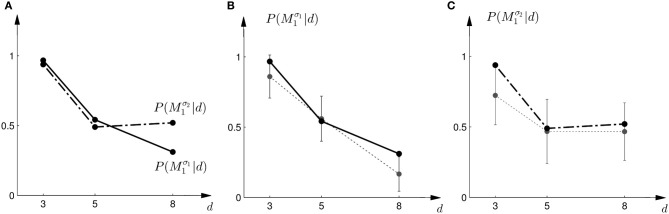
**Predictions and data: Bayes factors. (A)** Shows the predicted choice probabilities for choosing *M*_1_ as a function of the observed array width *d* under the assumption of a softmax choice rule. The solid line corresponds to the predicted choice probabilities for the first part of the study, the dash-dot line shows the predicted choice probabilities for the second part of the study, where the variance of *M*_1_ was increased. **(B)** Shows the predictions for the first part of the study (solid line) and the experimental results for three different widths *d* of the ambiguous feedback array. The dotted line shows the mean and standard deviation across subjects of the experimentally observed probabilities of choosing *M*^σ_1_^_1_. **(C)** Shows the predictions (dash-dot line) as well as the experimentally observed choice probabilities (dotted line represents mean with standard deviation error bars across subjects) for choosing *M*^σ_2_^_1_ in the second part of the study.

Once we have computed the model evidence for all possible observations and models, we can use it to make predictions about subjects' choice probabilities between the models. As we have equal prior probabilities *P*(*M*_*i*_) for the models in our experiment, the model class *M*_*i*_ that assigns a higher marginal likelihood *P*(*d*|*M*_*i*_) to the observation *d* is predicted to be preferred. Model selection is then determined by the *Bayes factor* (Kass and Raftery, 1993) between the two models, that is *P*(*d*|*M*_1_)/*P*(*d*|*M*_2_). Based on a softmax decision rule, we can then predict subjects' choice probabilities as

$$P(a=M_{1})=\frac{1}{1+e^{-\alpha \log\frac{P(d|M_{1})}{P(d|M_{2})}}},$$

where *a* = *M*_1_ implies moving up to choose model *M*_1_ and *a* = *M*_2_ implies moving down to choose model *M*_2_. We assumed α = 1 throughout. Thus, if the Bayes factor is larger than one, subjects should be more likely to choose Model 1. Conversely, if the Bayes factor is smaller than one, subjects should be more likely to choose Model 2. The choice probabilities resulting from the softmax rule are shown in Figure 7A. In the case of small variance σ_1_, this predicts that the probability of choosing model *M*_1_ should decrease with the increase of the uncertainty of the ambiguous feedback. In the case of large variance σ_2_, this predicts that the probability of choosing model *M*_1_ is very similar to the probability of choosing model *M*_2_ for medium and large feedback arrays. Especially for the large ambiguous feedback array of width *d* = 8 cm—the prediction implies that for the *same* stimulus the choice probabilities for selecting model *M*_2_ are very different depending on the complexity of model *M*_1_.

The comparison to the actual probabilities of model selection observed in the experiment are presented in Figures 7B,C. In line with the predictions shown in Figure 7A the probability of choosing model *M*^σ_1_^_1_ decreases with increasing width of the ambiguous feedback array for the first part of the experiment: for small array widths subjects preferred model *M*_1_, whereas for large ambiguous feedback arrays they preferred model *M*_2_. Similarly, the predictions explain the choice probabilities of the probe trials in the second part of the experiment, where for small array widths model *M*_1_ is preferred, and for larger array widths subjects are indifferent between the two models. The predictions achieved a negative log-likelihood of *L* = 1170 with respect to the data.

### Explanation 2: Bayesian policy inference

Instead of learning different prior distributions *P*(*s*|*M*_*i*_) over the shifts for the two models *M*_1_ and *M*_2_, subjects could directly learn optimal responses *P*(*a* = *M*_1_|*s*) to the shifts in the standard trials and a single prior *P*(*s*) over the possible shifts. They could learn, for example, that for small shifts they should mostly move to the upper target, that is *a* = *M*_1_, and for large shifts they should mostly move to the lower target, that is *a* = *M*_2_. When faced with an ambiguous stimulus in a probe trial, they could then integrate over the responses *P*(*a*|*s*) by considering all possible shifts weighted by the plausibility of each shift. This plausibility is given by the posterior distribution *P*(*s*|*d*) that results from inferring the underlying shift after observing an array of width *d*. The choice probability in the probe trial can then be computed by the integral

$$P(a=M_{1}|d)=\int dsP(s|d)P(a=M_{1}|s).$$

This choice rule has been previously proposed as a stochastic Bayesian rule for control in (Ortega and Braun, 2010) to solve adaptive control problems. In this framework it is assumed that a number of primitive strategies are known that are suitable for different environments. When knowledge of the environment is not available a probabilistic superposition of the primitive strategies results in a stochastic strategy that conforms with Bayesian statistics. In our experiment the different environments correspond to trials with different shifts. The basic strategies coping with these shifts could be learned in the standard trials together with a prior *P*(*s*) over all possible shifts. The unconditional prior *P*(*s*) is given by the superposition of the two conditional priors shown in Figure 2, that is $P(s)=\frac{1}{2}P(s|M_{1})+\frac{1}{2}P(s|M_{2})$. In the probe trials ambiguity is induced about the underlying shift. The possible underlying shifts can be inferred through the posterior *P*(*s*|*d*) that is given by *P*(*s*|*d*) ∝ *P*(*d*|*s*)*P*(*s*), with the same likelihood model *P*(*d*|*s*) as described in the previous section and displayed in Figure 4B.

**Figure 8:**
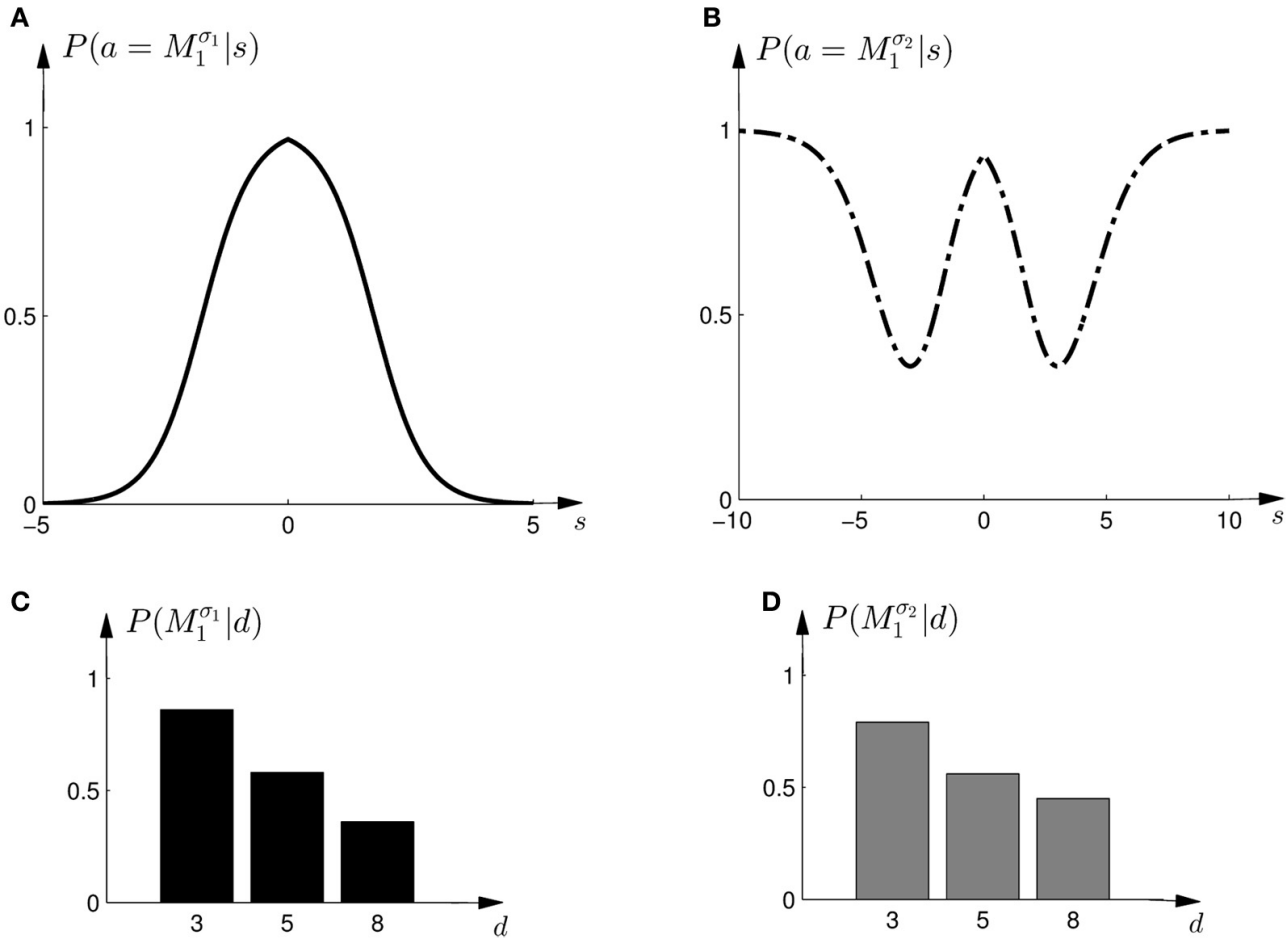
**Construction of predicted choice probabilities following Bayesian policy inference. (A)** Shows the fitted choice probabilities of selecting model *M*_1_ in standard trials when the shift *s* is known for a typical subject in the first part of the experiment. Model *M*_1_ is mostly selected for small shifts. **(B)** Shows same as panel **(A)** but for the second part of the experiment. **(C,D)** Show the predicted choice probability of selecting model *M*_1_ for this subject in a probe trial with observed array width *d*. The probe trial choice probabilities are obtained by a probabilistic superposition of the standard trial choice probabilities shown in panels **(A,B)**.

**Figure 9:**
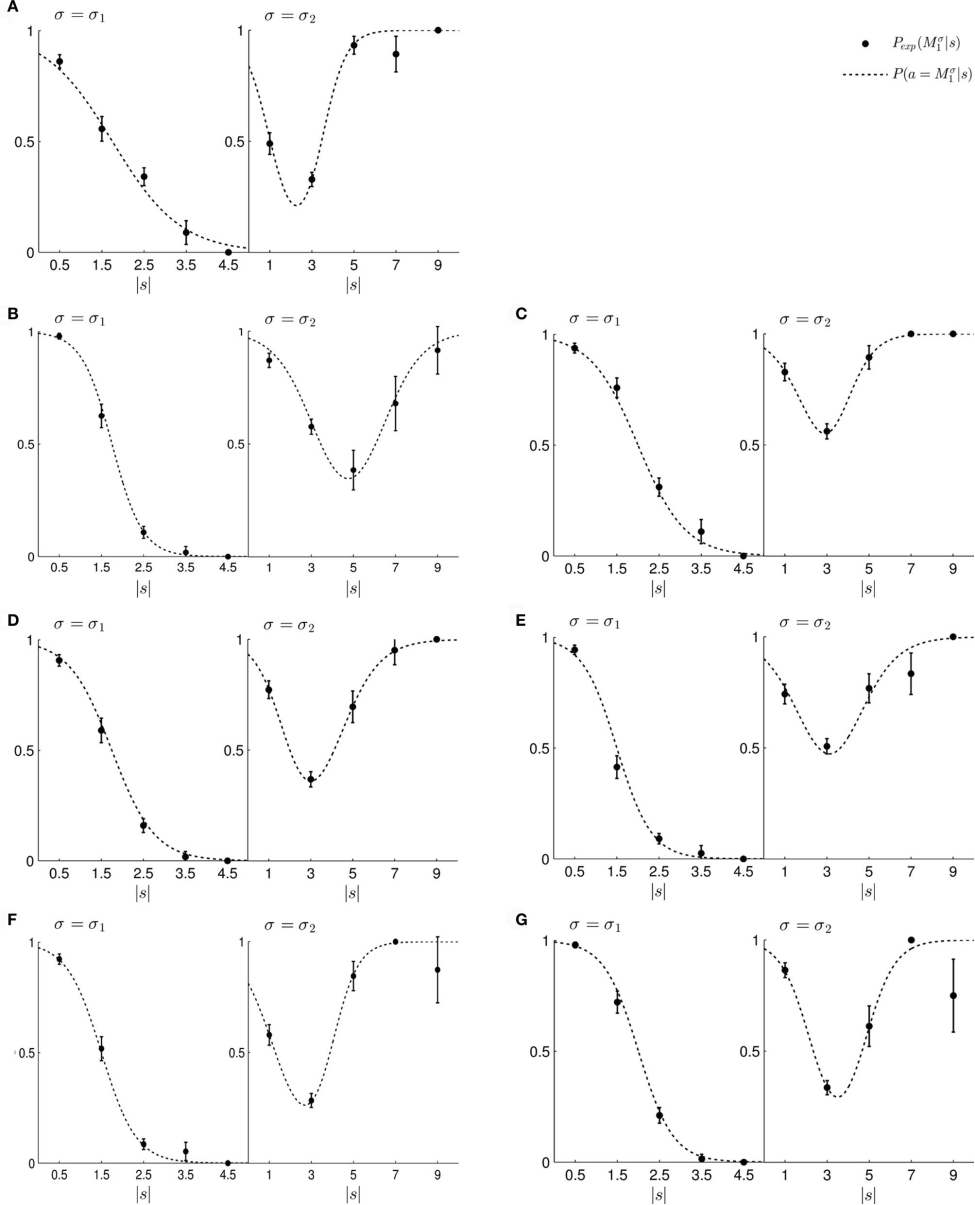
**(A–G)** Show the fitted standard trial choice probabilities of selecting model *M*_1_ for all seven subjects. The left panel shows performance during the first part of the experiment and the right panel shows performance during the second part of the experiment. In each panel, the filled circles show the subject's mean choice probability *P*(*M*^σ^_1_), given a particular shift *s* along with standard deviation error bars. The choice probabilities for negative shifts have been mapped to the corresponding positive shift. The dotted line shows the fitted response curve that lies closest to the observed choice probabilities.

To test this model, we first investigated subjects' choice behavior in standard trials. In particular, we examined subjects' probability *P*(*a* = *M*_1_|*s*) of choosing model *M*_1_ or *M*_2_ for different shifts *s*. The response curves *P*(*a* = *M*_1_|*s*) for a typical subject can be seen in Figures 8A,B. Panel (A) shows the response curve for the first part of the experiment, and Panel (B) shows the response curve for the second part of the experiment. In both cases subjects showed a high probability for choosing model *M*_1_ for small shifts. In the first part of the experiment this probability decreases for large shifts, implying the selection of model *M*_2_. In the second part of the experiment the probability of selecting model *M*_1_ decreases for larger shifts, but then increases again with very large shifts. These response curves are in agreement with the prior distributions shown in Figure 3, as in the first part of the experiment model *M*_1_ was only associated with small shifts, whereas in the second part of the experiment model *M*_1_ could also be associated with very large shifts. Learning the response functions *P*(*a* = *M*_1_|*s*) is therefore equivalent to learning the conditional priors *P*(*s*|*M*_*i*_). The fitted response curves for all subjects can be seen in Figure 9.

**Figure 10:**
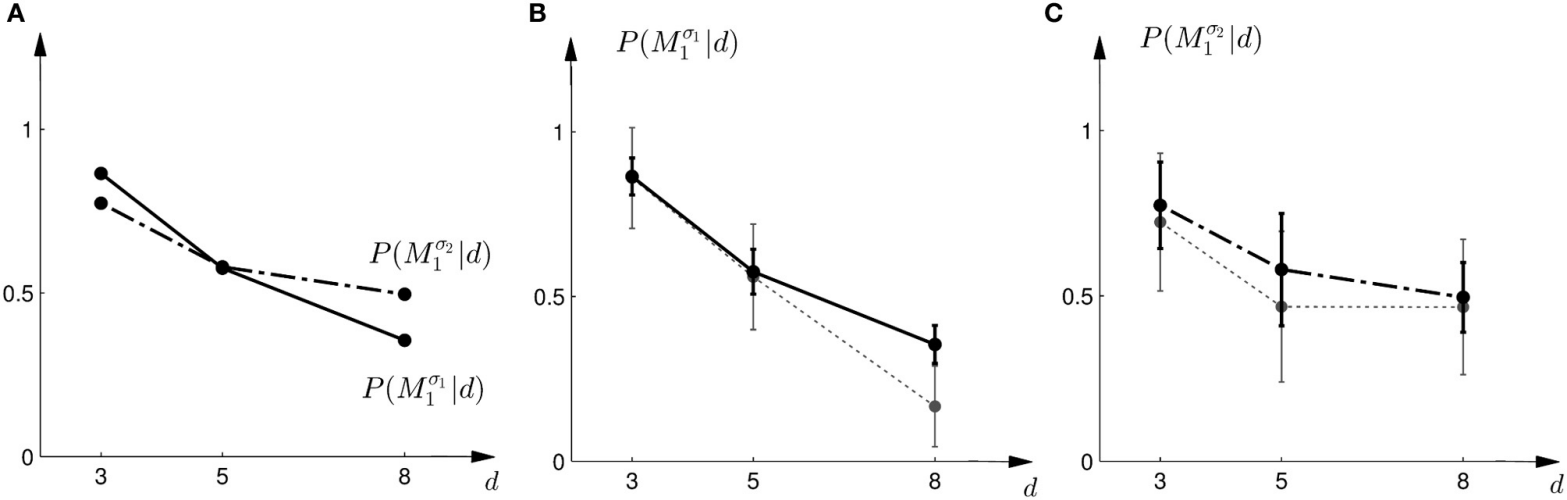
**Predictions and data: Bayesian policy inference. (A)** Shows the predicted choice probabilities for choosing *M*_1_ as a function of the observed array width *d* using response functions that have been fitted to the standard trial data. The solid line corresponds to the predicted choice probabilities for the first part of the study, the dash-dot line shows the predicted choice probabilities for the second part of the study, where the variance of *M*_1_ was increased. Since the response functions have been fitted individually per subject, the predictions show the mean across all subjects. The corresponding standard deviation error bars are shown in panels **(B,C)**. **(B)** Shows the predictions for the first part of the study (solid line) and the experimental results for three different widths *d* of the ambiguous feedback array. The dotted line shows the mean and standard deviation across subjects of the experimentally observed probabilities of choosing *M*^σ_1_^_1_. **(C)** Shows the predictions (dash-dot line) as well as the experimentally observed choice probabilities (dotted line) for choosing *M*^σ_2_^_1_ in the second part of the study.

In the probe trials the underlying shift is unknown and therefore the policy *P*(*a* = *M*_1_|*s*) cannot be applied directly, as it requires knowledge of the shift *s*. Using Bayesian policy inference, the action is then determined by a probabilistic superposition that is weighted by the posterior probabilities of the shifts. This superposition allows predicting directly the choice probabilities for the probe trials. The predicted choice probabilities are shown for a typical subject in Figures 8C,D. Panel (C) shows the subject's predicted probability of choosing model *M*_1_ for three different array widths *d* in the first part of the experiment. It can be seen that the choice probability decreases for large observed array widths. Similarly, Panel (D) shows the subject's predicted probability of choosing model *M*_1_ in the second part of the experiment. In this case the choice probability for model *M*_1_ is elevated for the small array width, but is close to one half for the two larger array sizes. The comparison to the actual choice probabilities of all subjects observed in the experiment are presented in Figure 10. The predictions achieved a total negative log-likelihood of *L* = 1097 with respect to the data.

### Explanation 3: the “average shift”-heuristic

**Figure 11:**
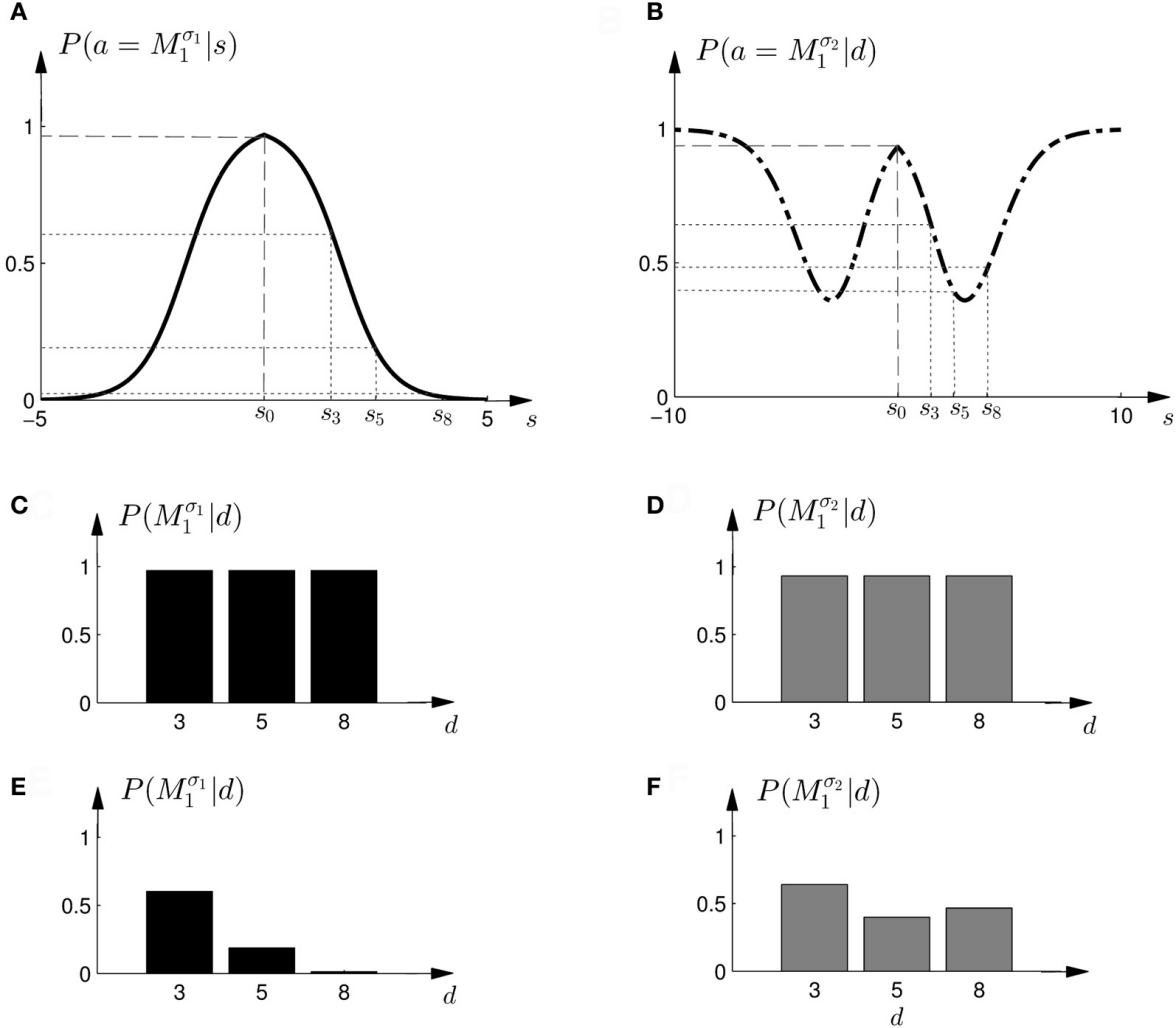
**Construction of predicted choice probabilities following heuristics. (A,B)** Show the fitted choice probabilities of selecting model *M*_1_ in standard trials of the same subject shown in Figure 8. For the “average shift”-heuristic the shift *s*_0_ is always assumed in the probe trials. For the “biggest shift”-heuristic the shift is always assumed to be the largest possible shift that depends on the width of the feedback array and ranges from *s*_3_ to *s*_8_. **(C,D)** Show the predicted choice probability of selecting model *M*_1_ for this subject in a probe trial with observed array width *d* under the “average shift”-heuristic. **(E,F)** Show the predicted choice probability of selecting model *M*_1_ for this subject in a probe trial with observed array width *d* under the “biggest shift”-heuristic.

The response curves *P*(*a*|*s*), that describe behavior in the standard trials in dependence of the observed shift *s*, could also be used for non-probabilistic heuristic strategies in probe trials. One such strategy could be to simply assume the average shift when faced with an ambiguous stimulus, which corresponds to the location in the middle of the cursor array in the probe trial—see Figures 11A,B. In this case the choice probabilities are determined by

$$P\text{(a=}M_{\text{1}}\text{|}d\text{) = }P(a=M_{\text{1}}\text{|}s\text{=0).}$$

**Figure 12:**
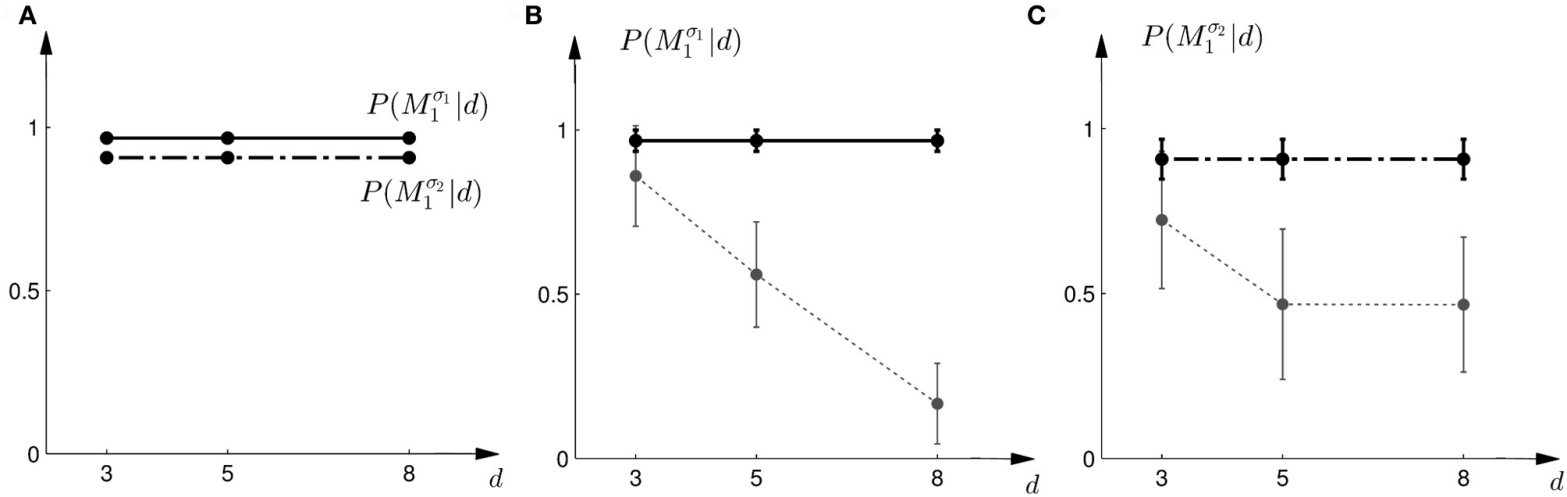
**Predictions and data: “average shift”-heuristic. (A)** Shows the predicted choice probabilities for choosing *M*_1_, assuming that subjects base their decision on the average shift when observing an array of width *d*. The predictions are based on response functions that have been fitted to the standard trial data. The solid line corresponds to the predicted choice probabilities for the first part of the study, the dash-dot line shows the predicted choice probabilities for the second part of the study, where the variance of *M*_1_ was increased. Since the response functions have been fitted individually per subject, the predictions show the mean across all subjects. The corresponding standard deviation error bars are shown in panels **(B,C)**. **(B)** Shows the predictions for the first part of the study (solid line) and the experimental results for three different widths *d* of the ambiguous feedback array. The dotted line shows the mean and standard deviation across subjects of the experimentally observed probabilities of choosing *M*^σ_1_^_1_. **(C)** Shows the predictions (dash-dot line) as well as the experimentally observed choice probabilities (dotted line) for choosing *M*^σ_2_^_1_ in the second part of the study.

However, this strategy predicts constant choice probabilities that do not vary with the observed array size. Moreover, this predicts that subjects should choose model *M*_1_ most of the time, as it explains shifts in the middle best—see the prediction for a typical subject in Figures 11C,D. This prediction is in clear contradiction to the observed choice probabilities that change depending on the feedback array width. Figure 12 shows the predictions of the “average shift”-heuristic compared to the actual choice probabilities of all subjects. The predictions of the “average shift”-heuristic achieved a total negative log-likelihood of *L* = 2689 with respect to the data.

### Explanation 4: the “biggest shift”-heuristic

Another non-probabilistic heuristic that could be employed based on the response curves of the standard trials, is to assume always the largest possible shift for any given cursor array in the probe trial. Accordingly, the location of the assumed shift would correspond to the edge of the array with total width *d*, such that the edge corresponds to the half-width *d*/2. The corresponding choice probabilities are determined by

$$P(a\text{=}M_{\text{1}}\text{|}d\text{) = }P(a=M_{\text{1}}\text{|}s=d\text{/2).}$$

**Figure 13:**
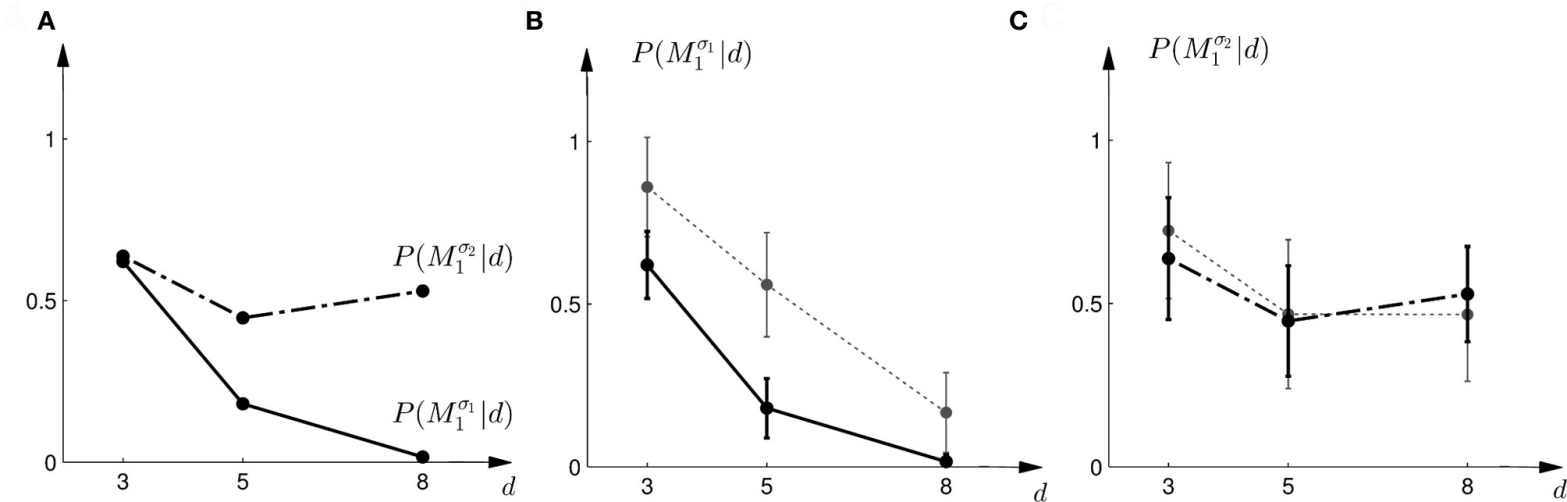
**Predictions and data: “biggest shift”-heuristic. (A)** Shows the predicted choice probabilities for choosing *M*_1_, assuming that subjects base their decision on a shift equal to the largest value that lies within the array of width *d*. The predictions are based on response functions that have been fitted to the standard trial data. The solid line corresponds to the predicted choice probabilities for the first part of the study, the dash-dot line shows the predicted choice probabilities for the second part of the study, where the variance of *M*_1_ was increased. Since the response functions have been fitted individually per subject, the predictions show the mean across all subjects. The corresponding standard deviation error bars are shown in panels **(B,C)**. **(B)** Shows the predictions for the first part of the study (solid line) and the experimental results for three different widths *d* of the ambiguous feedback array. The dotted line shows the mean and standard deviation across subjects of the experimentally observed probabilities of choosing *M*^σ_1_^_1_. **(C)** Shows the predictions (dash-dot line) as well as the experimentally observed choice probabilities (dotted line) for choosing *M*^σ_2_^_1_ in the second part of the study.

The predictions of the “biggest shift”-heuristic can be seen in Figures 11E,F for a typical subject. As in the two Bayesian models, the predicted choice probability of model *M*_1_ decreases with increasing array width for the first part of the experiment. For the second part of the experiment the “biggest shift”-heuristic predicts a slightly increased probability of choosing model *M*_1_ for the small array width and almost indifferent choice probabilities for the two larger array widths. Figure 13 shows the actual choice probabilities of all subjects compared to the predictions. While the “biggest shift”-heuristic predicts the right trend in the first part of the experiments, it considerably underestimates the actual choice probabilities. The predictions for the second part of the experiment lie within the standard deviation of the experimental data. The predictions of the “biggest shift”-heuristic achieved a total negative log-likelihood of *L* = 1321 with respect to the data.

### Explanation 5: the “halfway shift”-heuristic

The probe trial shifts are grossly underestimated by the “average shift”-heuristic and overestimated by the “biggest shift”-heuristic. Accordingly the choice probabilities for model *M*_1_ are either too high or too low, especially in the first part of the experiment. We therefore considered a “halfway shift”-heuristic that would always assume a shift halfway between the middle and the edge of the cursor array. Accordingly, the choice probabilities of the “halfway shift”-heuristic lie inbetween the extremes of the other two heuristics

$$P(a=M_{\text{1}}\text{|}d)\;=\;P\text{(}a=M_{\text{1}}\text{|}s=d\text{/4) .}$$

**Figure 14:**
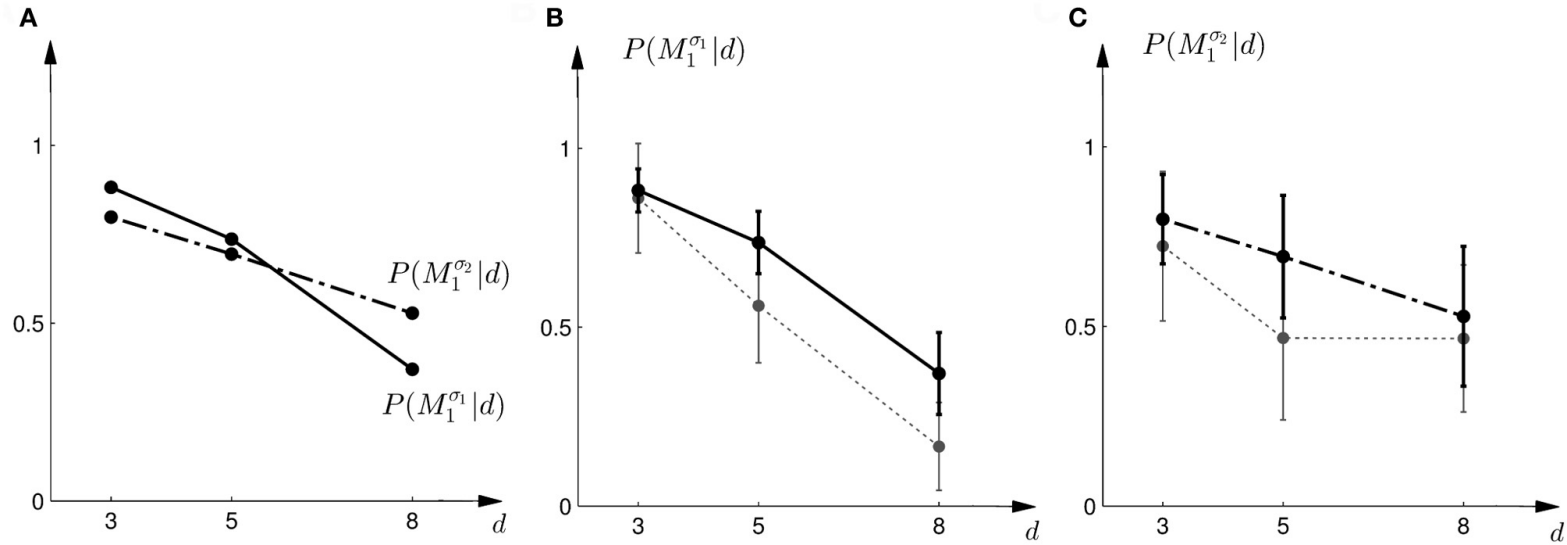
**Predictions and data: “halfway shift”-heuristic. (A)** Shows the predicted choice probabilities for choosing *M*_1_, assuming that subjects base their decision on a shift equal to half of the largest value that lies within the array of width *d*. The predictions are based on response functions that have been fitted to the standard trial data. The solid line corresponds to the predicted choice probabilities for the first part of the study, the dash-dot line shows the predicted choice probabilities for the second part of the study, where the variance of *M*_1_ was increased. Since the response functions have been fitted individually per subject, the predictions show the mean across all subjects. The corresponding standard deviation error bars are shown in panels **(B,C)**. **(B)** Shows the predictions for the first part of the study (solid line) and the experimental results for three different widths *d* of the ambiguous feedback array. The dotted line shows the mean and standard deviation across subjects of the experimentally observed probabilities of choosing *M*^σ_1_^_1_. **(C)** Shows the predictions (dash-dot line) as well as the experimentally observed choice probabilities (dotted line) for choosing *M*^σ_2_^_1_ in the second part of the study.

Figure 14 shows the actual choice probabilities of all subjects compared to the predictions of the “halfway shift”-heuristic. While the error bars of the experimental data and the theoretical curves overlap, it can be seen that the heuristic generally overestimates the choice probability of choosing model *M*_1_. The predictions of the “halfway shift”-heuristic achieved a total negative log-likelihood of *L* = 1203 with respect to the data.

## Discussion

We designed a three-dimensional visuomotor integration experiment where we could distinguish between parameter variables and model variables, such that the parameter variable was represented by lateral visuomotor shifts in one dimension and the model variable was represented by two targets in the other dimension that were associated with different distributions over the shifts. In particular, we designed probe trials that did not require subjects to compensate these shifts, such that the shift variable could be “integrated out” when they reported their belief about the underlying model class. This allowed us to directly compare subjects' choice probabilities to the selection probabilities predicted by five different schemes of model selection: Bayesian model selection based on Bayes factors, Bayesian policy inference over response curves that were fitted to the standard trials, and three non-probabilistic heuristics that were also based on the standard trial response curves. We found that the Bayesian model selection procedures explained our data best, whereas the three heuristics were worse in explaining choice behavior in the probe trials. By testing two sets of distributions over the shifts, for which the observed model selection probabilities agreed with the predictions of two different Bayesian model selection procedures, we achieved a proof of concept for this experimental paradigm.

The experimental paradigm differs from previous sensorimotor paradigms on Bayesian integration (Körding and Wolpert, 2004) in two important ways. First, by introducing a third dimension to the task we can simultaneously induce uncertainty over two random variables, one of which can represent a parameter variable and the other one a model variable. Previous studies (Körding and Wolpert, 2004) have shown Bayesian integration in visuomotor tasks where only uncertainty over parameters was investigated, meaning that subjects had to infer visuomotor shifts which were drawn from a particular distribution. It was shown that subjects combined information about the prior distribution of these shifts together with noisy sensory feedback in order to obtain an optimal estimate of the shift. By varying the reliability of the sensory feedback, the authors could show that subjects weighted prior and feedback in a Bayesian way—giving less weight to the feedback if reliability of the feedback was low. As there was only one distribution over shifts, the authors could not test for Bayesian model selection in their experiment. By introducing the third dimension for the model variable, we could therefore naturally extend their paradigm.

Second, we developed a paradigm where we can separately query the model and parameter variables from subjects. We achieved this by enlarging the horizontal size of the targets in probe trials, such that lateral corrections, that are used to report the shift parameter, become obsolete. Moreover, we clamped horizontal movements in probe trials with a force channel to ensure that only the model variable is reported. These precautions are necessary, in particular if we assume that subjects report maximum a posteriori estimates, because the maximum of a joint distribution max_*s,M*_ *P*(*s, M*|*d*) is not necessarily the same as the maximum of the marginal distribution max_*M*_ ∑_s_ *P*(*s, M*|*d*). This asymmetry is one of the most important problems when designing a sensorimotor paradigm for model selection, in which the model variable has to be queried non-verbally.

As subjects were instructed verbally at the beginning of the experiment about the relationship between the cursor shifts and the two targets, the question arises in how far cognitive processes might have played a role during the experiment. We instructed subjects about the cursor-target relationship to speed up and simplify the learning process, as we were not primarily interested in standard trial performance, but in probe trial behavior. Subjects could use these instructions as a good first guess to discriminate between the two targets. Importantly, the verbal instructions relevant for standard trials did not eliminate any of the ambiguity faced in probe trials that needed to be resolved during the movement. In this sense, our task can be conceived as a generalization of previous sensorimotor tasks (Körding and Wolpert, 2004). Nevertheless we cannot rule out that cognitive processes played a role in the perception of the ambiguous stimuli during the probe trials and the subsequent discrimination between the two models, as cognitive and sensorimotor processes are often intertwined. However, even cognitive processes have been previously shown to be consistent with Bayesian inference.

In cognitive science and perceptual learning hierarchical Bayesian inference over model classes and model parameters has been previously investigated in a number of studies (Tenenbaum et al., 2006; Körding et al., 2007; Sato et al., 2007; Holyoak, 2008; Kemp and Tenenbaum, 2008, 2009; Tenenbaum et al., 2011) In particular, (Körding et al., 2007) have studied integration vs. segregation of audio-visual stimuli in human subjects—which included inference over the two models *M*_1_ and *M*_2_: (*M*_1_) there is only one source for both stimuli with a location parameter *s* and (*M*_2_) there are two different sources for the two stimuli with location parameters *s*_visual_ and *s*_audio_. To specifically look into the probabilities of model selection they modeled data from a similar previous experiment (Wallace et al., 2004), where subjects were asked to report their perception of unity. In contrast, our experimental paradigm allows for reporting model selection without the need of explicitly asking subjects verbally and without them being aware that one of the task dimensions represents a parameter variable and the other a model variable.

We compared five different strategies that could explain subjects' model selection probabilities in probe trials. The two Bayesian explanations had the lowest negative likelihood and therefore explained the choice probabilities in the probe trials best. However, there are important differences between the two Bayesian explanations. The first explanation explicitly computes the marginal likelihoods and uses these likelihoods as a discriminative variable. This requires the probabilistic representation of the conditional priors *P*(*s*|*M*), the prior over the models *P*(*M*), and the likelihood model *P*(*d*|*s*). The optimal strategy is then determined over the marginal likelihood that results from an integration of these distributions. In contrast, Bayesian policy inference results from a stochastic superposition of given policies, which in our case correspond to the model selection probabilities in standard trials when the visuomotor shift is known. In probe trials, the model selection probabilities can then be determined by an integral over these standard trial policies. If subjects' choice behavior was non-stochastic we could easily distinguish between these two possibilities, as decisions based on the marginal likelihood bear no intrinsic stochasticity—we imposed it here through the softmax-function—and decisions resulting from the probabilistic superposition of standard trial policies would always be stochastic. Given the error bars on our data and the fact that real decision-making processes are always somewhat noisy, it is hard to distinguish between the two processes, even though the Bayesian policy inference achieved the lowest negative likelihood—compare Figures 7, 10.

We also examined three simple heuristics and tested in how far they might be able to explain the observed choice probabilities in probe trials. We investigated heuristics that did not consider any probabilistic representation of the task. In particular, we were investigating in how far standard trial policies could be harnessed to construct heuristics for the probe trials. A first heuristic assumed that subjects would always use the standard trial policy associated with a zero shift right in the middle of the ambiguous cursor array in the probe trial (the “average shift”-heuristic). A second heuristic assumed that subjects would always use the standard trial policy associated with the largest possible shift at the edge of the ambiguous cursor array in the probe trial (the “biggest shift”-heuristic). A third heuristic was a mixture between the two, always using the standard trial policy associated with a shift halfway between the middle and the edge of the cursor array. Especially, the first two heuristics provided very poor explanations of the choice behavior, because they either systematically under- or overestimated the probability of choosing one of the models. The “halfway shift”-heuristic achieved a negative log-likelihood value that was only slightly higher than model selection with Bayes factors, but the mismatch in the fits still seemed to be systematically biased—see Figure 14. More importantly, the question remains why any of these heuristics would be formed and applied. As subjects did not receive any performance feedback in the probe trials, they could not have learned the heuristics from trial and error.

Bayesian methods are typically used in two different ways in psychophysical studies. They can simply be used as techniques to analyze the data or they can be interpreted as processes that might take place in a “Bayesian brain” that tries to make sense of the world around it. Here we used different Bayesian and non-Bayesian explanations to describe subjects' choice behavior in a model selection task. This does not necessarily have any implications as to which precise algorithm the brain might use to achieve this behavior. In fact, there are a number of methods that have been suggested for the problem of model selection: the Akaike Information Criterion (AIC) (Akaike, 1974), the Schwarz or Bayesian Information Criterion (BIC) (Schwarz, 1978), minimum description length (MDL) (Rissanen, 1978), Bayes factors (Kass and Raftery, 1993; MacKay, 2003), structural risk minimization (Vapnik, 1995), and regularization methods (Bishop, 2006). Model selection criteria like AIC and BIC can be considered as approximations to Bayesian model selection, but also MDL, regularization and complexity measures in statistical learning theory can be related to the consideration of prior probabilities in Baysian model selection (MacKay, 2003). Finally, how model selection is achieved by neurons in the brain is subject to neurophysiological investigation.

A key capability of biological organisms is to cope with an uncertain environment. Uncertainty has many sources. It can originate from noise in the nervous system (Faisal et al., 2008), but also from uncertainty that arises in the face of ambiguous stimuli. In dealing with uncertainty, Bayesian statistics have proven to be a powerful and unifying framework not only in cognitive sciences, but also in sensorimotor tasks (Körding and Wolpert, 2006) and neural computation (Knill and Pouget, 2004; Doya, 2007; Orban and Wolpert, 2011). In particular, hierarchical Bayesian models for inference and control might allow modeling a variety of learning processes on multiple levels of abstraction (Haruno et al., 2003). Our task design provides a means to study such hierarchical integration in the context of sensorimotor control.

## Materials and methods

### Participants

Three female and four male participants were recruited from the student population of the University of Tübingen. The study was approved by the local ethics committee and all participants were naive and gave informed consent. The local standard rate of eight Euros per hour was paid for participation in the study.

### Materials

**Figure 15:**
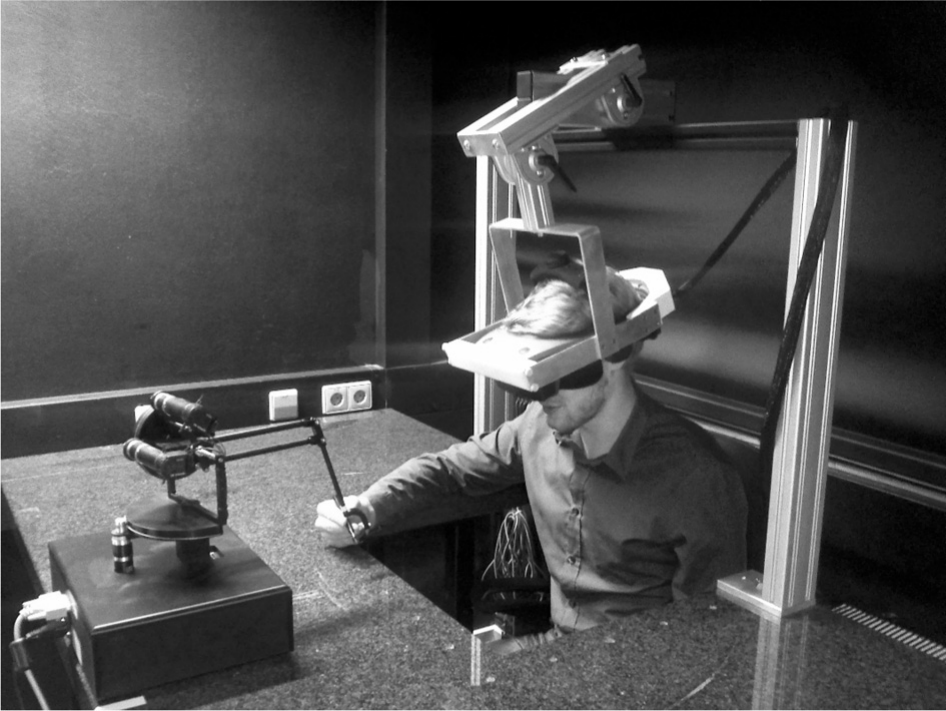
**Photograph of the experimental apparatus.** The subject operates a Sensable^®^ Phantom^®^ Premium 1.5 High Force manipulandum and receives stereoscopic 3D visual feedback through an NVIS^®^ nVisor ST50 head-mounted display.

We used a virtual reality setup consisting of a Sensable^®^ Phantom^®^ Premium 1.5 High Force manipulandum for tracking participants' hand movements in three dimensions and an NVIS^®^ nVisor ST50 head-mounted display (HMD) for creating stereoscopic 3D virtual reality—see Figure 15. Movement position and velocity were recorded with a rate of 1 kHz. To prevent very fast movements, the manipulandum was operated with a weak isotropic viscous force field of $\vec{f}=\alpha I_{3\times 3}\dot{\vec{x}}$, where $\alpha =0.04\frac{Ns}{\text{cm}},\;I_{3\times 3}$ is the identity and $\dot{\vec{x}}$ is the three-dimensional velocity vector.

### Experimental design

A model selection problem can be characterized by a bivariate distribution *P*(*s, M*) over a continuous random variable *s* and a binary random variable *M*, where *s* plays the role of the model parameter and *M* plays the role of the model. To study model selection in a sensorimotor context, we designed a 3D visuomotor task where participants had to move a cursor from a start position to one of two targets, referred to as upper and lower target in the following—see Figure 1. During the movement, the horizontal position of the cursor was shifted, and the shift was generated by one of two possible statistical models *M* ∈ {*M*_1_, *M*_2_} with 50:50 probability. Importantly, subjects were not informed about the 50:50 probability. Each target corresponded to a model *M*—the upper target corresponded to *M*_1_ and the lower target corresponded to *M*_2_. The correct target was the one whose corresponding model *M* actually generated the observed shift in any particular trial. The shifts were generated by first sampling a model *M* and then sampling a shift from the shift-distribution *P*(*s*|*M*). Since shifts were generated probabilistically, any shift could in principle be generated by either model, however, with different probabilities. There was only brief sensory feedback of the shifted cursor during the movement. Participants had to use this feedback together with their knowledge of the previously learned statistical models *P*(*s*|*M*) to not only infer the shift *s*, but also the model *M*. In these *standard trials*, participants reported their belief *P*(*s, M*), where the shift *s* was indicated by a compensatory horizontal movement when hitting a target, and the belief about the model *M* was reported by choosing one of the two targets.

In order to test for model selection in case of feedback uncertainty, participants also experienced *probe trials*, in which they only reported their belief *P*(*M*) = ∫ *dsP*(*s, M*) about the model *M*. This was achieved by increasing the width of the targets to cover the whole horizontal workspace, such that no horizontal compensatory movements were necessary in these trials. During the movement in probe trials, sensory feedback was briefly shown in shape of arrays consisting of uniformly and densely sampled rectangles that represented all the possible cursor locations—see Figure 1B. Each probe trial had one of three possible feedback array sizes (small, medium and large) that occurred equi-probably. Since the size of the feedback array constrained the uncertainty about the possible shifts *s*, Bayesian model selection required participants to “integrate out” different intervals of the parameter *s* when deciding on the model *M* by choosing either the upper or lower target.

### Trial setup

Each participant performed two parts of the experiment consisting of 500 trials each. Before starting the experiment, participants were informed about the relation between observing the horizontal cursor shift and selecting one of the two targets. To start them off in the first part of the experiment, they were told that small shifts would be often associated with the upper target and larger shifts mostly with the lower target—but they were also instructed that they should use the first 100 training trials for learning this relationship precisely. Similarly, they were told for the second part of the experiment that small and very large shifts would be often associated with the upper target and medium large shifts mostly with the lower target. The initial 100 trials of each of the two sessions were standard trials only. To keep participants motivated, the hit ratio (in percent of the standard trials presented so far) was displayed. In the following 400 trials standard and probe trials were intermixed. For the probe trials, subjects were instructed that there would be a whole array of little cursors any of which could be the true cursor and that again they would have to decide which one was the correct target just like in the standard trials, but this time without knowing for sure which cursor was the correct one. The probability of presenting a probe trial was 0.45 if the previous trial was a standard trial and 0 if the previous trial was a probe trial. The second block of 500 trials was identical, only the probability distribution over shifts of model *M*_1_ was broadened to investigate the effect on the model selection process.

### Experimental prior distributions

Each model induced a different prior probability density *P*(*s*|*M*) over the horizontal shifts *s*. In part one of the study, the two models were a Gaussian and a bimodal mixture of Gaussians:

$$M_{1}:\;P(s|M_{1}^{\sigma 1})\;=N(0,\;1\;{\text{cm}}^{2})$$

$$M_{2}:\;P(s|M_{2})\;=\frac{1}{2}N(-2.5\;\text{cm},0.25\;{\text{cm}}^{2})\;+\frac{1}{2}N(2.5\;\text{cm},0.25\;{\text{cm}}^{2}).$$

In part two of the study, the same distributions were used, only the standard deviation of *P*(*s*|*M*_1_) was increased such that

$$M_{1}:\;P(s|M_{1}^{\sigma 2})\;=N(0,16\;{\text{cm}}^{2})$$

$$M_{2}:\;P(s|M_{2})\;=\frac{1}{2}N(-2.5\;\text{cm},0.25\;{\text{cm}}^{2})\;+\frac{1}{2}N(2.5\;\text{cm},0.25\;{\text{cm}}^{2}).$$

The prior probability of both models was always $P(M_{1})=P(M_{2})=\frac{1}{2}.$ A plot of the prior distributions is shown in Figure 3.

### Experimental procedure: standard trials

After hearing a beep, participants initiated a reaching movement by controlling a *cursor* (red sphere, radius 0.4 cm) from a start position (gray sphere, semi-transparent, radius 0.9 cm) to one of two target blocks (yellow cuboids, height 5 cm, width 2 cm)—see Figure 1A. One of the target blocks was in the upper half of the workspace, the other target block was in the lower half—with a distance of 2 cm in-between. Both target blocks were presented at a depth of 18.5 cm with respect to the start position. Once the cursor had left the start position, it was invisible and an additive random shift was applied to the cursor position. The shift was drawn from a distribution *P*(*s*|*M*) once *M* had been sampled from *P*(*M*). The correct target was determined by the sampled *M*, that is the upper target was correct if *M* = *M*_1_ and the lower target was correct if *M* = *M*_2_. While the cursor was invisible during the movement, after a movement depth of 5.5 cm visual feedback (red rectangle, width 0.8 cm, height 0.3 cm) of the shifted cursor position was displayed for 100 ms. When the movement exceeded a depth of 18.5 cm the trial ended. If the cursor was in-between the two targets without touching either of them, the trial continued until one of the targets was chosen. For hitting a target, the cursor had to at least touch the target block. When participants hit the correct target, a high-pitch beep was played. When participants hit the wrong target or missed the correct target, a low-pitch beep was played. In either case the incorrect target disappeared. At the end of the reach, the shifted cursor position was shown. If movement was still in progress after 2 s, the trial was aborted and had to be repeated.

### Experimental procedure: probe trials

In contrast to standard trials, the target width of the two targets was increased to 20 cm in probe trials, thereby covering the entire horizontal workspace. Crucially, this made any compensatory movements in the horizontal direction obsolete and reduced the task to a binary model selection problem that only required choosing either the upper or lower target. To further discourage horizontal compensatory movements in probe trials, we generated a “force tunnel” that did not allow left/right deviations from the middle of the workspace, but only up/down and forward/backward movements. Since sideward movements were not necessary in probe trials, the impact of the tunnel force was barely noticeable and most participants reported that they did not notice it at all when interviewed after the experiment.

Probe trials also started with a beep, after which participants initiated a reaching movement to one of the two targets (yellow cuboids, height 5 cm, width 20 cm)—see Figure 1B. At a movement depth of 5.5 cm visual feedback was displayed for 100 ms. However, in contrast to the standard trials, feedback was not shown as a little rectangle representing the shifted hand position, but as an array of multiple same-sized rectangles that were sampled simultaneously and uniformly from one of three possible horizontal intervals: [−1.5, 1.5], [−2.5, 2.5], and [−4.0, 4.0] cm. The little rectangles had 0.8 cm width and there were 4, 7, and 10 little rectangles, respectively shown for small, medium and large bar size at any one time frame. The probability of showing one of the array sizes was one third. Participants were informed that this array indicated all possible cursor positions and that the true cursor was at one of the many possible positions seen in the array. Since sideward deviations were impossible due to the tunnel force and vertical deviations carried no information with respect to possible shifts, the arrays were always centered in the workspace both horizontally and vertically. In order to make participants understand that the shift was sampled uniformly from the cursor array, at the end of the movement, after participants had chosen one of the two targets, a cursor position was drawn from the uniform distribution over the shown interval and displayed. However, no visual or auditive feedback was given to indicate whether the correct target was hit or not. The three possible widths for the interval (small: 3 cm, medium: 5 cm, and large: 8 cm) induced an increasing amount of uncertainty about possible shifts. In the probe trials we could therefore investigate how these different amounts of uncertainty with respect to the parameter *s* affected the selection of the model *M*, when the parameter *s* was not reported and therefore could be “integrated out.”

### Modeling

For the two Bayesian explanations of choice behavior in probe trials, we used the following observation model. Crucially, in our experiment observing an array of width *d* ≥ 0 is the same for both models *M*_1_ and *M*_2_ and therefore the likelihood model only depends on the shift variable *s*, such that

$$P(d|s)=\{\begin{array}{cc} \frac{1}{\frac{d_{\max}}{2}-|s|} & \text{if }d\geq \text{2|}s\text{| and }d\leq d_{\max}\\ 0 & \text{otherwise}, \end{array}$$

where *d*_max_ represents the maximum possible array width. In our experiment *d*_max_ = 8 cm. This observation model implies that for any given shift *s*, the array size cannot be smaller than *s*, since it must contain *s*, and the array size cannot exceed the maximum size *d*_max_. All array sizes in-between have equal probability. After observing array size *d*, Bayes' rule allows us to infer the posterior over both the shift and the model

$$P(s,M|d)=\frac{P(d|s)P(s|M)P(M)}{\sum_{M}\int dsP(d|s)P(s|M)P(M)}.$$

The Bayes factor can be derived from this posterior by realizing that *P*(*d*|*M*) = *P*(*M*|*d*) and *P*(*M*|*d*) = ∫ *dsP*(*s, M*|*d*). In case of the Bayesian policy inference the posterior *P*(*s*|*d*) can be derived from the joint posterior by realizing that *P*(*s*|*d*) = ∑_*M*_ *P*(*s, M*|*d*).

In line with Bayesian policy inference the posterior *P*(*s*|*d*) is used for the probabilistic superposition of the standard trial policies *P*(*a* = *M*_1_|*s*) such that the choice probability in probe trials is given by *P*(*a* = *M*_1_|*d*) = ∫ *dsP*(*s*|*d*)*P*(*a* = *M*_1_|*s*). The standard trial policies *P*(*a*|*s*) were fitted to the data as follows. First, all standard trials were sorted into five equidistant bins depending on the magnitude of the shift in each trial. For the first part of the experiment the five bins were [0, 1], [1, 2], [2, 3], [3, 4], and [4, 5] cm. For the second part of the experiment the five bins were [0, 2], [2, 4], [4, 6], [6, 8], and [8, 10] cm. The relative frequencies of choosing model *M*_1_ in these bins was fitted by a sigmoid psychometric function

$$P(a=M_{1}|s)=1-\frac{1}{1+e^{\frac{-s+\zeta }{\theta }}}$$

for the first part of the experiment and a two-partite sigmoid function

$$P(a=M_{1}|s)=1-\frac{1}{1+e^{\frac{-s+\gamma }{\delta }}}+\frac{1}{1+e^{\frac{-s+\kappa }{\tau }}}$$

for the second part of the experiment. The free parameters ζ, θ, γ, δ, κ, τ were fitted by minimizing square error. The fits can be seen in Figure 9.

### Conflict of interest statement

The authors declare that the research was conducted in the absence of any commercial or financial relationships that could be construed as a potential conflict of interest.
